# Supplementary material for: Speciation Analysis Highlights the Interactions of Auranofin with the Cytoskeleton Proteins of Lung Cancer Cells
Source: Pharmaceuticals (Basel). 2022 Oct 19;15(10):1285. doi: 10.3390/ph15101285 (PMC9610265; doi:10.3390/ph15101285)
Supplement: Supplementary file 1 [file pharmaceuticals-15-01285-s001.zip › Supplementary_metarial_figures_S1-S3_rev.pdf]

Supplementary electronic material

Figure S1. **(a)** Sankey's graphs showing the distribution of platinum (a characteristic component of cisplatin) in three fractions obtained by LLE extraction carried out for adherent (AC) and non-adherent (DC) MRC-5 and A-549 cells. **(b)** SEC-ICP-MS chromatograms obtained for lysates of cells exposed to cisplatin. The chromatogram of the control sample for MRC-5 cells matched the chromatogram obtained for the control sample for A-549 cells. Chromatograms for the control sample spiked with cisplatin also consisted of one peak observed at the same retention time.

Figure S2. Cell cycle analysis for auranofin in comparison to cisplatin.

Figure S3. A wound healing test performed for epithelial cells exposed to auranofin and cisplatin.

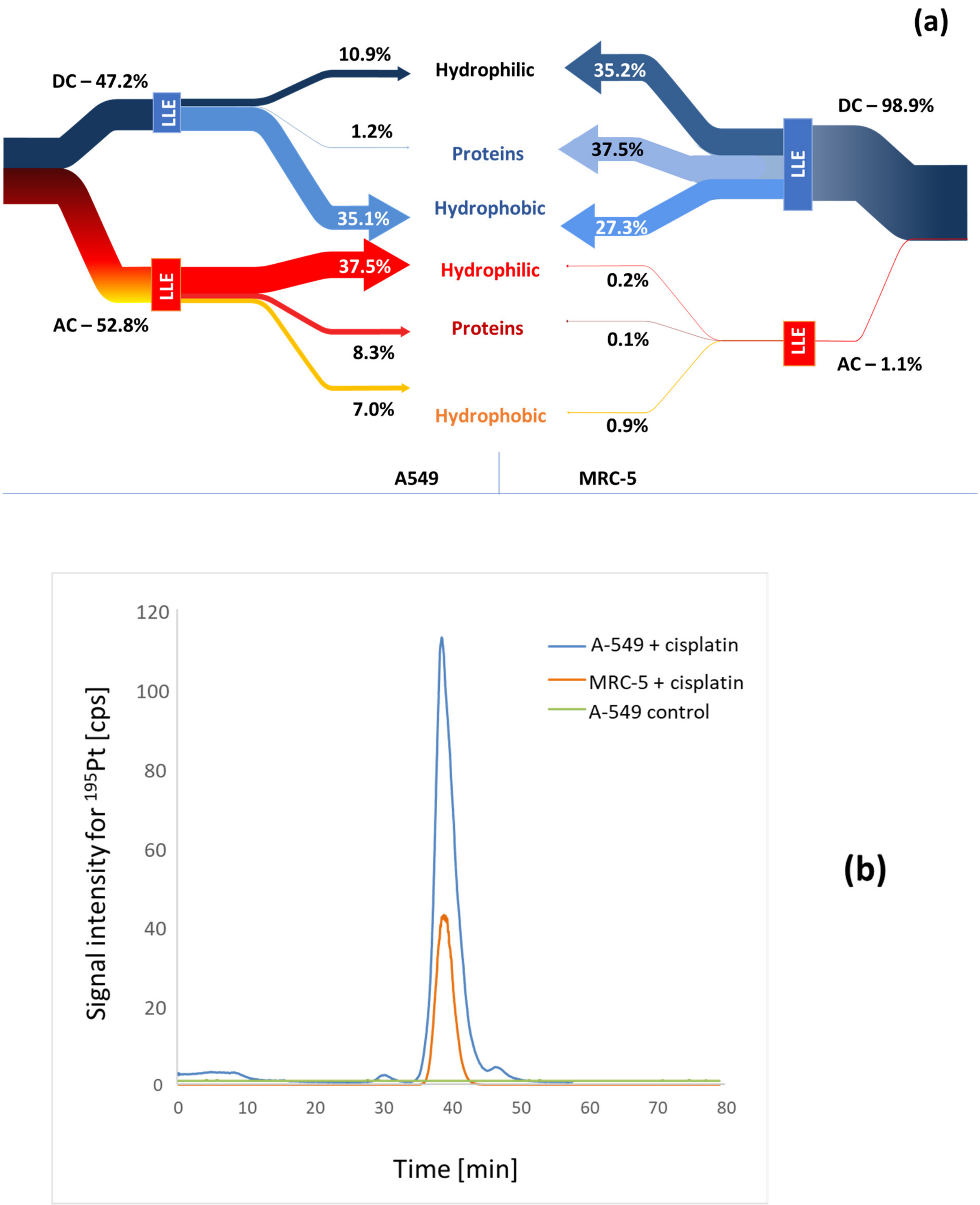

Figure S1. **(a)** Sankey's graphs showing the distribution of platinum (a characteristic component of cisplatin) in three fractions obtained by LLE extraction carried out for adherent (AC) and non-adherent (DC) MRC-5 and A-549 cells. **(b)** SEC-ICP-MS chromatograms obtained for lysates of cells exposed to cisplatin. The chromatogram of the control sample for MRC-5 cells matched the chromatogram obtained for the control sample for A-549 cells. Chromatograms for the control sample spiked with cisplatin also consisted of one peak observed at the same retention time.

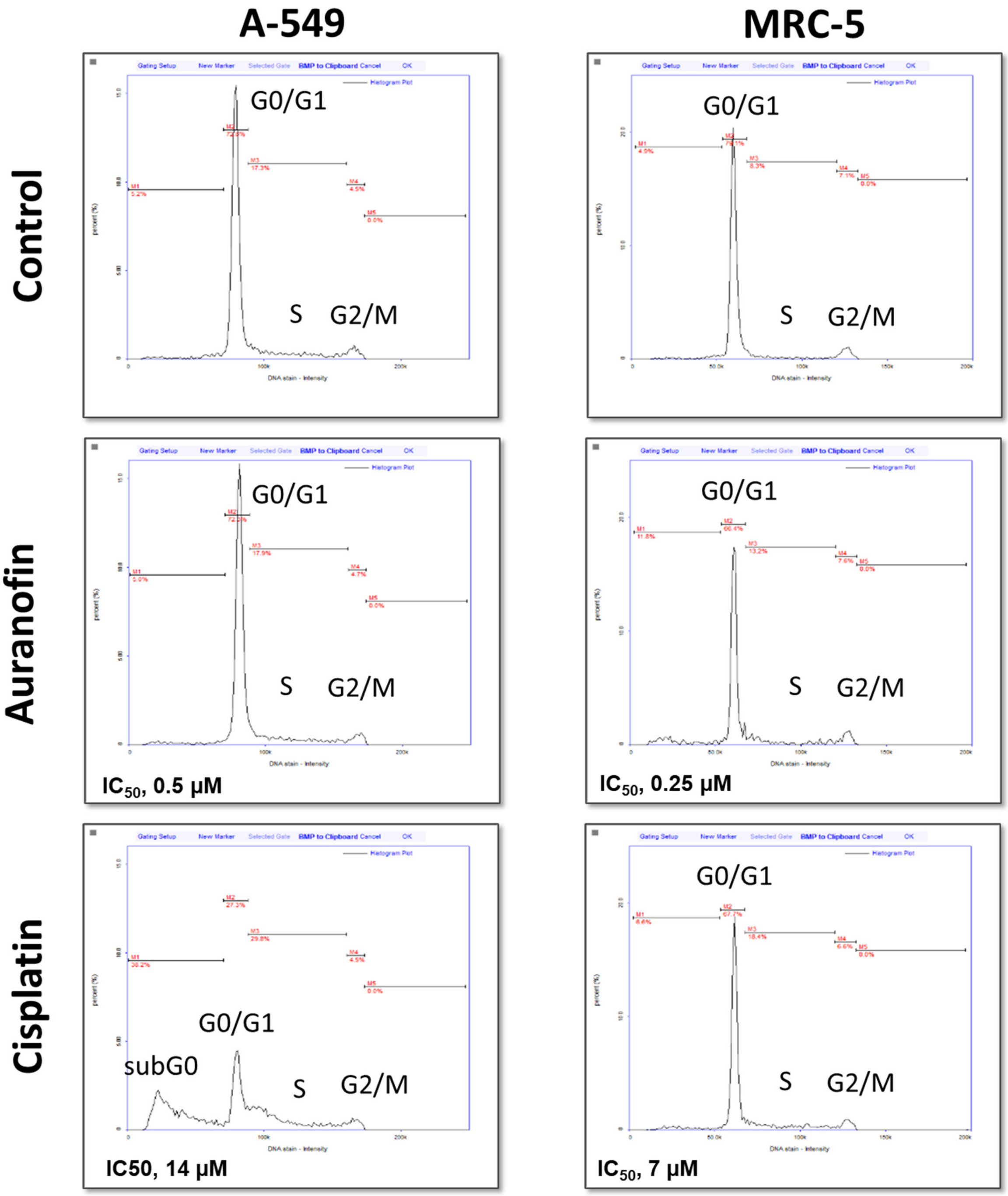

Figure S2. Cell cycle analysis for auranofin in comparison to cisplatin.

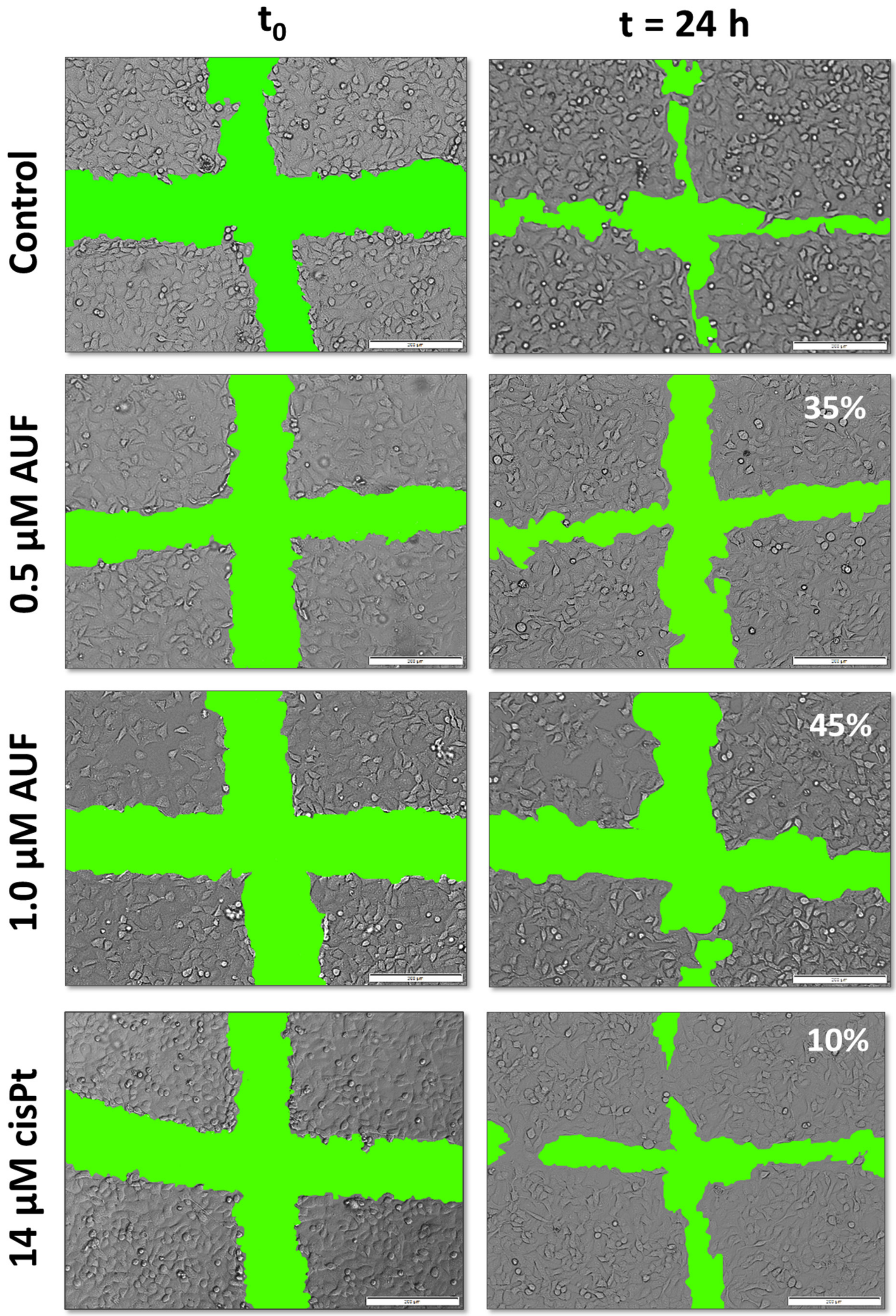

Figure S3. A wound healing test performed for epithelial cells exposed to auranofin and cisplatin
